# Ligature of Left Carotid Artery for Hemorrhage Following Kiotomy

**Published:** 1880-08

**Authors:** Hadar Liden, Herman Mynter

**Affiliations:** Med. Lic.


### Translations.

---

### LIGATURE OF LEFT CAROTID ARTERY FOR HEMORRHAGE FOLLOWING KIOTOMY, BY HADAR LIDÉN, MED. LIC.

TRANSLATED FROM SWEDISH BY HERMAN MYNTER, M. D.

As a dangerous hemorrhage after kiotomy, performed lege-artis, is so seldom met with that prominent surgeons deny the possibility, the author considers it his duty to publish the following case:

November 28th, 1879; was consulted by a lady who complained of coryza and of cough, which seemed to take its origin in the throat. By inspection of the throat both tonsils were found hypertrophied to such a degree that they almost touched each other, the left one being the larger. Kiotomy was proposed and the patient consented, although with reluctance.

The left tonsil was first removed, being caught with a forceps and cut off with a probe-pointed bistoury from below upwards along its attachment to the wall of the pharynx. There was no unusual bleeding immediately afterwards. The patient gargled the throat with cold water, but the bleeding did not stop, increasing little by little, so that the patient continually coughed up coagulated dark colored blood. No arterial bleeding was discovered. The bleeding continued for three hours in spite of the application of ice, perchlorid of iron, and solid nitrate of silver, increased continually, and became more arterial in color. The pulse was 130, weak and commenced to be irregular; as the patient was evidently failing, the left common carotid artery was ligated without narcosis, and the bleeding stopped then perfectly. The case progressed slowly but favorably, and the patient left the hospital in about three weeks; the ligature was removed several months afterward. The coryza and cough had then almost disappeared.

The author considers the bleeding occasioned by abnormal distribution of the arteries. He does not think it possible that arteria maxilaris interna was cut by the kiotomy, as the blood was dark colored and never came in spurts, but more like a parenchymatous bleeding. Possibly there was a deep-seated angioma, some branches of which were cut by the kiotomy. For this speaks that the patient always had complained of strong pulsation over the left carotid artery. Homophily had never occurred in her family, and she had never had any symptoms of that nature.—*Hygiea.*

